# Cow’s milk allergy: towards an update of DRACMA guidelines

**DOI:** 10.1186/s40413-016-0125-0

**Published:** 2016-11-15

**Authors:** Alessandro Fiocchi, Lamia Dahda, Christophe Dupont, Cristina Campoy, Vincenzo Fierro, Antonio Nieto

**Affiliations:** 1 Division of Allergy, University Department of Pediatrics, Pediatric Hospital Bambino Gesù, Rome, Vatican City Italy; 2 Service d’Explorations Fonctionnelles Digestives Pédiatriques, Hôpital Necker, Université Paris-Descartes, 149, rue de Sèvres, 75015 Paris, France; 3 Department of Paediatrics, Centre of Excellence for Paediatric Research EURISTIKOS, School of Medicine, University of Granada, Avda. De Madrid 11, 18012 Granada, Spain; 4 Department of Paediatrics, University of Granada, Avda. de la Investigación 11, 18016 Granada, Spain; 5 Pediatric Pulmonology & Allergy Unit, Children’s Hospital La Fe, Valencia, Spain

**Keywords:** Cow’s milk allergy, Infants, DRACMA guidelines

## Abstract

**Background:**

In 2010, the diagnosis and treatment of IgE-mediated CMA were systematized in a GRADE guideline.

**Objectives & methods:**

After 6 years, the state of the knowledge in diagnosis and treatment of CMA has largely evolved. We summarize here the main advances, and exemplify indicating some specific points: studies aimed at better knowledge of the effects of breastfeeding and the production of new special formulae intended for the treatment of CMA. The literature (PubMed/MEDLINE) was searched using the following algorithms: (1) [milk allergy] AND diagnosis; (2) [milk allergy] AND [formul*] OR [breast*], setting the search engine [6-years] time and [human] limits. The authors drew on their collective clinical experience to restrict retrieved studies to those of relevance to a pediatric allergy practice.

**Results:**

Several clinical studies did address the possibility to diagnose CMA using new tools in vitro and in vivo, or to diagnose it without any evaluation of sensitization. Some studies also addressed the clinical role of formulae based on milk hydrolysates, soy, or rice hydrolysates in the treatment of CMA. Many studies have elucidated the effects of selective nutrients in breastfed infants on their immunologic and neurologic characteristics.

**Conclusions:**

Evidence-based diagnostic criteria should be identified for non-IgE-mediated CMA. Debate is ongoing about the best substitute for infants with CMA. In particular, Hydrolyzed Rice Formulae have been widely assessed in the last six years. In the substitute choice, clinicians should be aware of recent studies that can modify the interpretation of the current recommendations. New systematic reviews and metanalyses are needed to confirm or modify the current DRACMA recommendations.

## Background

Six years have passed since the World Allergy Organization ﻿(WAO)﻿ Diagnosis and Rationale for Action against Cow’s Milk Allergy (DRACMA) guidelines systematized the diagnosis, prognosis, and treatment of allergy to cow's milk proteins (CMA) [1]. Since then, advances have been made in all the fields covered by the guidelines. In diagnosis, the possibility of interpreting the pathology by molecular test has improved [2, 3]. Studies have been made on the use of patch tests [4] and of new tests, such as the Basophil Activation Test, in some clinical situations [5]. The possibility to use clinical scores to diagnose non IgE-mediated CMA has been proposed [6], and is now the subject of intense debate [7, 8]. New studies have clarified the natural history of the disease [9, 10] and the possible role of microbiome in shaping it [11]. The successful introduction of baked products into the diet before tolerance development to unprocessed milk changed our daily practice, and it is now debated if this offers a new possibility to modify it [12, 13]. However, the field where we made the greatest strides is the replacement therapy. Many new formulas have been developed, entering the everyday use. These formulas are aimed to not only improve their nutritional value, nutrient balance, and allergologic safety, but also include functional components, some also found in formulas for normal children, some specific for infants with CMA.

In this article, we will offer an overview of CMA in its development over the last six years. We will pay particular attention to the advances in the field of therapy, looking at the unmet needs indicated in the DRACMA guidelines, and to the way they have been dealt with (if not met) by the scientific community. To this end, we cite as an example some of the topics that may become the subject of revision in the new guidelines.

## 2010–16: many more reasons for considering breastfeeding as the first substitute in CMA

Faced with an infant with CMA, the pediatrician must dictate an avoidance regimen. This will include a substitute; the best is – of course – breastfeeding with a mother’s diet free of milk products [1]. Breastfeeding is strongly recommended as the preferred way of infant feeding [14]. Compared to formula-fed infants, the breastfed show:

- Better brain development
- Different growth patterns
- Different nutritional status
- Better immunologic system development & immune responses
- Different gut microflora
- Fewer infections, of shorter duration.

Despite the fact that formulae are modeled after breastmilk, the human milk composition maintains its unique characteristics. It contains a series of inimitable molecules with potential immune modulating activities. Examples comprise:

- maternal antibodies, including anti-idiotypic antibodies, able to sustain and regulate immune cell populations through a priming of fetal and neonatal cells;
- cytokines (TGF-β2, IL-10, thymic stromal lymphopoietin) and chemokines, influencing the development of allergy and atopic diseases;
- hormones and growth factors, influencing the maturation of the infant gut and of the associated lymphoid tissues;
- PUFAs, nucleotides, glycoproteins, oligosaccharides and microRNA, able in turn to exert immune functions.

Probably also for these reasons breastfeeding has been shown to influence a series of outcomes, including the establishment of gut microbiota, the prevention of overweight and obesity, the development of immunoallergic parameters and the neural development.

This last aspect is being actively investigated, and in the last few years offers us some new acquisitions that can be of interest in the general management of CMA. Breastfed babies display important structural differences in the brain anatomy compared with those that received infant formula: for instance, they present a longer corpus callosum, a higher ganglyothalamic ovoid diameter [15], a higher cortical thickness in parietal lobules [16]. Probably related to these effects, breastfeeding positively influences cognitive development and general intelligence [14].

From studies in cohorts of non-allergic infants it is known that the neural programming displays some ‘windows of plasticity’, during which environmental, nutritional, and microbiological factors may influence the brain function, generating different behavioral competence trajectories [17]. Many animal studies have focused on the effects of nutrition on brain development demonstrating that changes in dietary nutrients can alter brain morphology as well as its biochemical functions. Before the year 2010, however, much of the evidence from human studies was retrospective. Then epidemiological birth cohort studies indicated that folate, n-3 fatty acids, iodine and iron administered in pregnancy may influence the brain development in healthy children [18]. In particular, from the ALSPAC cohort we know that:

- Maternal seafood intake during pregnancy of less than 340 g per week is associated with increased risk of their children being in the lowest quartile for verbal intelligence quotient (IQ), compared with mothers who consumed more than 340 g per week [19]
- Low maternal seafood intake was also associated with increased risk of suboptimum outcomes for prosocial behaviour, fine motor skills, communication, and social development scores. For each outcome measured, the lower the intake of seafood during pregnancy, the higher the risk of suboptimum developmental outcome [19]
- Iodine deficiency during pregnancy is associated with negative cognitive outcomes [19, 20].

After birth, the more implicated nutrients in the global development of infants are protein supply, PUFAs, Vitamins B12, C, A and D, iron, iodine, choline, zinc, selenium, and copper. Independently or in combination, their nutritional availability may influence cognitive performance behavior [21]. Other studies failed to identify positive effects of breastfeeding on early life intelligence and cognitive growth from toddlerhood through adolescence [22], while an improved performance in intelligence of breastfed children was found at age 30 [23]. Taken together, the recent human studies indicate that the association among breastfeeding and improved performance in intelligence tests is not casual [24].

As a case in point, essential fatty acids play a central role in brain development of infants: humans can synthesize saturated and monounsaturated fatty acids but cannot synthesize the n-3 and the n-6 families of PUFA. The parent fatty acids of these families, alpha linolenic acid (18 carbons, three double bonds with the first double bond in the n-3 position, C18:3n-3, ALA) and linoleic acid (C18:2n-6, LA) are essential fatty acids and must be present in the diet. ALA is converted to eicosapentaenoic acid (C20:5 n-3, EPA) then to docosahexaenoic acid (C22:6n-3, DHA), while LA is converted to arachidonic acid (C20:4n-6, AA). DHA is a critical component of cell membranes, especially in the brain and retina. AA is both a membrane component and a precursor to potent signaling molecules, the prostaglandins and leukotrienes. The human milk always contains both AA and DHA, while in the past infant formulae had neither. Interventional studies failed to find evidence that prenatal fish-oil (and folic acid) supplementation may influence the cognitive development of children at 6.5 y of age, but a high DHA in maternal erythrocytes at delivery was associated with a Mental Processing Composite Score higher than the 50th percentile in the offspring [25]. Also, associations of maternal LC-PUFA status with child emotional and behavioral problems were found in an epidemiologic study [26]. Nowadays, special formulae for the treatment of CMA are not in line with these characteristics of HM (Table 1).

**Table 1:** General characteristics of infant formulas for CMA

CMA infant formula composition	
Energy	Similar to HM
Proteins	Within normal recommended ranges, but CMP are hydrolysate, or whole proteins different than human milk proteins; some supplemented with lysine, threonine or tryptophan
Fats	Only 15 % have α-linolenic acid in similar amounts than HM; 31 % have more linoleic acid than HM; 46 % do not include DHA; one includes 25 % palmitic acid in beta position.
Carbohydrates	70 % of special formulae are without lactose; all have a content of carbohydrates higher than HM
Micronutrients	Fe ≤ than in HM (risk of iron-deficiency). Content of other minerals should be reviewed considering other factors.
Vitamins A, E, D	Need to be reviewed the doses depending on other factors *(>25 % of children consumed <2/3 of the RDI of Ca, Vitamins D and E)*.
Nucleotides	77 % have nucleotides
Choline	Big variability in choline levels between different formulae.
Taurine	92 % have taurine
Carnitine	92 % have carnitine
Prebiotics	15 % include FOS/GOS
Probiotics	8 % include probiotics

**Fig. 1:**
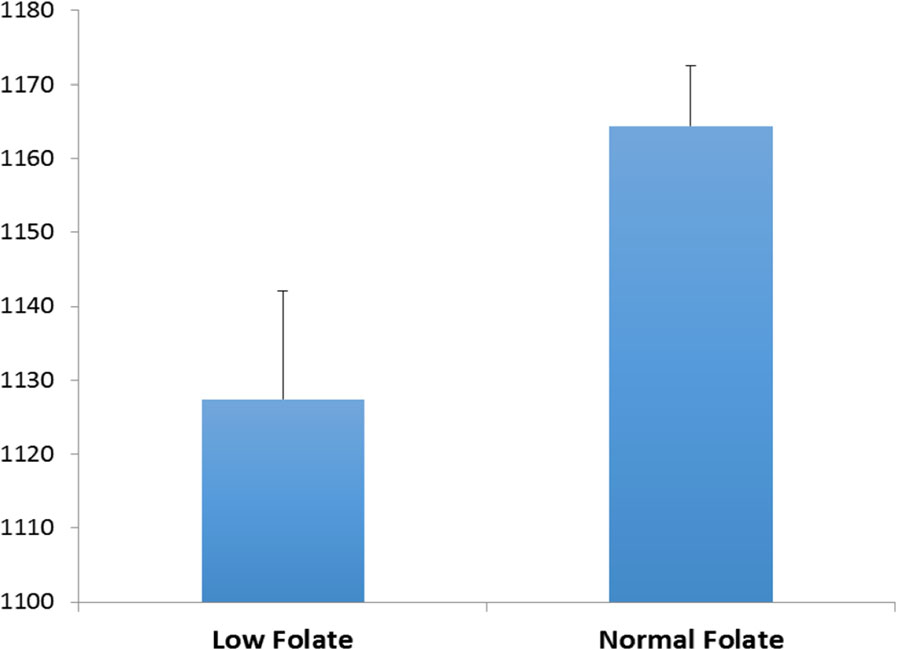
Total brain volume of children born to mothers with inappropriate and appropriate assumption of folic acid supplements in the first trimester of pregnancy

Another important component of breast milk is folic acid; its appropriate availability at the onset of pregnancy is associated with brain volume (Fig. 1). In children with low maternal folate levels, the head grows 0.1 mm per week less than in the controls [27]. This may translate in 1.9 million neurons and 1.9 billion synapses less per week. Low maternal folate status during early pregnancy was also found associated with a higher risk of emotional and behavioral problems in the offspring [28]. The use of prenatal folic acid supplements around the time of conception has been associated with a lower risk of autistic disorder [29]. Human milk provides sufficient folate intake, essential for normal growth and brain development; heat treatment in the breastmilk banks may critically reduce its amount [30].

Breastfeeding also influences the gut microbiota. Its establishment soon after birth is conditioned by factors as the type of delivery (passage through the birth canal vs. caesarean section), socioeconomic and climatic environment (born in developed vs. developing countries), and immune system development during pregnancy, antibiotic treatments, and contacts with parents, siblings and hospital staff [31]. Dietary factors (breast vs. formula feeding) are of prominent importance in this context.

**Fig. 2:**
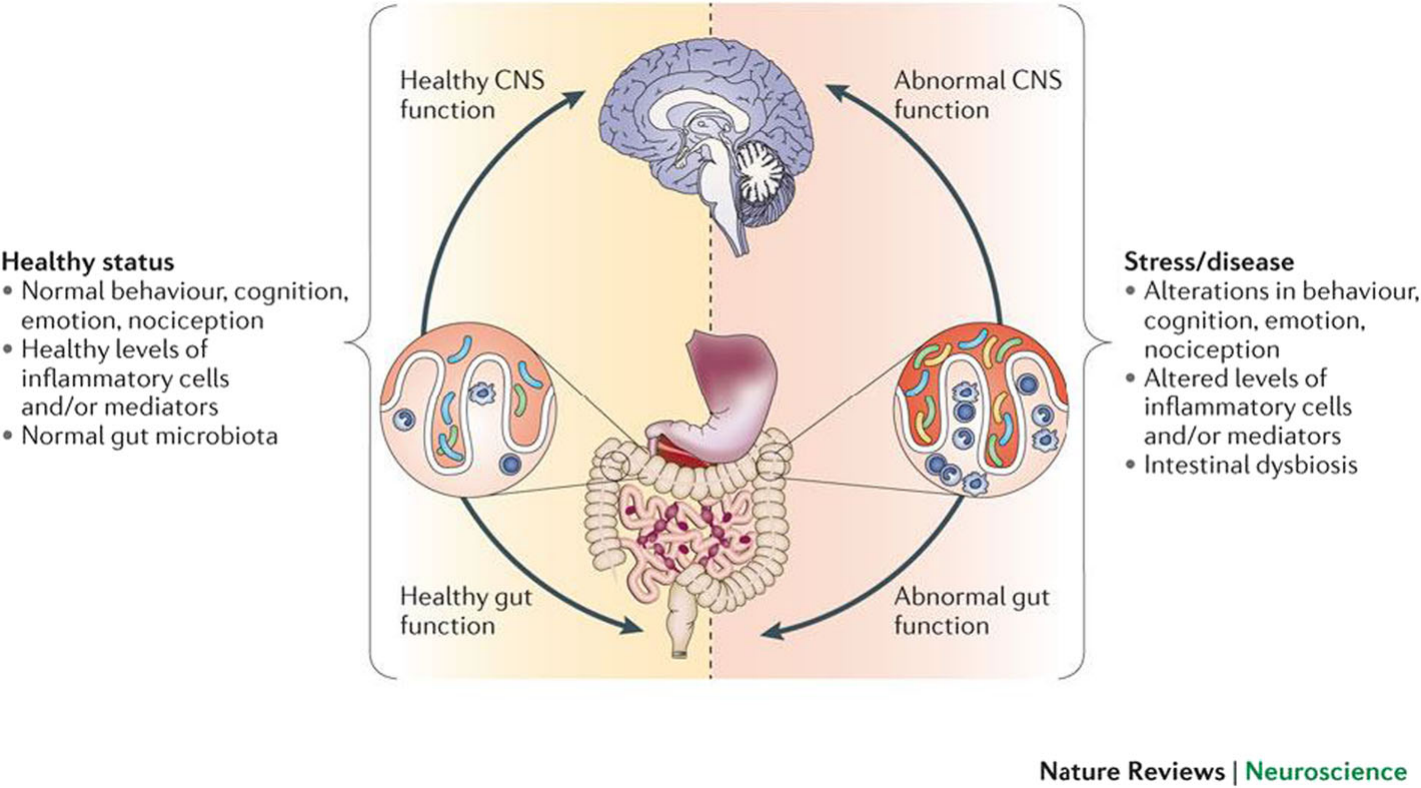
The gut-brain axis: interactions between microbiota and CNS functions

The gut microbiota as a major topic of research interest in biology has increased in recent years . Studies are assessing the influence of variations in the composition of the gut microbiota on diseases, ranging from inflammation to obesity. Accumulating data now indicate that the gut microbiota also communicates with the CNS — possibly through neural, endocrine and immune pathways — and thereby influences brain function and behavior. Studies in germ-free animals and in animals exposed to pathogenic bacterial infections, probiotic bacteria or antibiotic drugs suggest a role for the gut microbiota in the regulation of anxiety, mood, cognition and pain [32]. It is now generally accepted that a stable gut microbiota is essential for normal gut physiology and contributes to appropriate signaling along the gut–brain axis and, thereby, to the healthy status of the individual, as shown on the left-hand side of Fig. 2. The right-hand side of the figure indicates how intestinal dysbiosis can adversely influence gut physiology, leading to inappropriate gut–brain axis signaling and associated consequences for CNS functions and resulting in disease states. Conversely, stress at the level of the CNS can affect gut function and lead to perturbations of the microbiota [33]. Thus, the emerging concept of a microbiota–gut–brain axis suggests that modulation of the gut microbiota may be a tractable strategy for developing novel therapeutics for complex CNS disorders.

Of course, all these activities of breastfeeding are mediated through epigenetic activities of the diet, especially during prenatal and early postnatal life. Diets high in choline, methionine, folate, vitamin B6 and vitamin B12 increase DNA and histone methylation altering gene expression and generating permanent changes in development [34]. Early-life nutritional exposures, therefore, can act on the development of asthma, allergy, and obesity through epigenetic mechanisms [35].

## From breastmilk to special formulae for children with CMA

Patients with cow’s milk allergy and their breastfeeding mothers must strictly avoid cow’s milk and cow’s milk protein-based products. Particularly in young children, a well-balanced diet with sufficient intake of calcium and other essential nutrients must be warranted. When non-breastfed, the staple food for infants with CMA are special infant formulae. In an era when the benefits of breastfeeding are better known, their use poses some questions from a nutritional point of view:

- Are the apparently reassuring effects on growth pattern of extensively hydrolyzed formulae (eHF), rice hydrolyzed formulae (RHF), or amino acid formulae (AAF) maintained over time?
- Have babies fed eHF, RHF, soy formulae (SF) or AAF long-term effects on neurodevelopment similar to those breastfed?
- If not, which are the effects associated to eHF, RHF, SF or AAF feeding on neurodevelopment, in relation to the different protein in the infant formula composition respect to standard cow’s milk infant formula?
- How does the use eHF, RHF, SF or AAF influence the epigenetic process in the brain?
- How does the “brain-body loop” work in children fed with eHF, RHF, SF or AAF?
- Are eHF, RHF, SF or AAF modifying the development of senses?
- Is the gut of the baby with CMA fed with eHF, RHF, SF or AAF functioning the same? Which will be the long-term consequences?

To assess these points, we must consider some differential characteristics of infant formulas for CMA vs human milk and standard infant formulas for healthy babies (Table 1). Taking into account these differences, most of the studies have been focused on growth [36–38].

Safety has also been evaluated on clinical symptoms (crying score, regurgitation score, stools, urticaria, eczema, respiratory symptoms, [39]) and some biochemical parameters as amino acid profile, plasma total protein, albumin, prealbumin, calcium, magnesium, and alkaline phosphatase [37]. All these studies have been reassuring on the nutritional effects of special formulae. However, we are far from understanding the role of all nutrients. The data published so far report short-term assessments; we need more data on the long-term follow-up of infants who were fed the new infant formulas to fully understand the role of these formulas and the functional compounds which are being added to them on these parameters [40].

## Controversies on the dietary management of CMA

With all these limitations, when faced with a non-breastfed infant with CMA the pediatrician must suggest a substitution formula. The DRACMA guidelines clearly suggest eHF as the first-line treatment for the majority of situations (Table 2). RHF are considered equivalent in the countries where they are available, and AAF are suggested only in severe clinical situations or in non-responder patients. In general, eHFs are nutritionally adequate and well tolerated by children allergic to cow’s milk and other foods, but their main drawbacks are a bitter taste [41], cost (2–3 times vs. standard formulae) and their potential to cause anaphylaxis. On the other hand, some specific sequences of the peptides in eHF which could possess potential immunomodulatory capacities, and, according to recent suggestions, could lead to active tolerance induction [42]. RHF are considered a second-line resource due to unavailability universally. Where available, RHF can be considered instead of eHF. AAFs are safe but are exorbitantly expensive (6–8 times the cost of eHFs) and not widely available [43]. Thus, which factors should the pediatrician take into account in choosing the right substitute? We develop here some simple considerations about the pros and cons of the different types of formulae, starting with the definition of food allergen.

**Table 2:** Choosing the appropriate substitute formula in different presentations (original source: DRACMA guidelines [1])

Clinical presentation	1st choice	2nd choice	3rd choice
Anaphylaxis	AAF^a^	eHF^e, d^	SF
Immediate gastrointestinal allergy	eHF^d, b^	AAF^f^/SF^g^	
Food protein-induced enterocolitis syndrome (CMAES)	AAF	eHF^c^	
Asthma and rhinitis	eHF^d, b^	AAF^f^/SF^g^	
Acute urticaria or angioedema	eHF^d, b^	AAF^f^/SF^g^	
Atopic dermatitis	eHF^d, b^	AAF^f^/SF^g^	
Gastroesophageal reflux disease (GERD)	eHF^b^	AAF	
Allergic eosinophilic oesophagitis	AAF		
Cow’s milk protein-induced enteropathy	eHF^d, b^	AAF	
Constipation	eHF^b^	AAF	Donkey milk^i^
Severe irritability (colic)	eHF^b^	AAF	
CM protein-induced gastroenteritis and proctocolitis	eHF^b^	AAF	
Milk-induced chronic pulmonary disease (Heiner’s syndrome)^h^	AAF^f^	SF	eHF

^a^Recommendation 7.1

^b^Recommendation 7.2

^c^If AAF refusal

^d^Subject to local availability, RHF can be considered instead than eHF (7.4)

^e^Subject to a negative SPT with the specific formula (panel recommendation)

^f^AAF if a relatively high value on avoiding sensitization by SF and/or a low value on resource expenditure are placed

^g^SF if a relatively low value on avoiding sensitization by SF and/or a high value on resource expenditure are placed

^h^This suggestion attributes a high value on avoiding exposure to even residual antigenic cow’s milk proteins

^i^Based on reports from one case series

## Milk allergens and eHF

Children allergic to cow’s milk are not allergic to the milk as a whole, but to some of its proteins; and neither to the protein itself, but to some specific parts able to attach specific IgE, called epitopes. Thus, proteins are composed of a more or less long chain of amino acids, and only some parts of this chain are able to attach specific IgE [44]. Cow’s milk contains several proteins, some of which are considered major allergens, some minor ones, while others have been rarely associated with reports of clinical reactions. Caseins and whey proteins of cow’s milk are listed in Table 3. Their epitopes can be of two types. When a longitudinal sequence of AAs placed in a given order is able to attach IgE, this is called a ‘sequential epitope’. However, in other cases this particular sequence is fictitious, because it is not in fact a lineal sequence of AAs, but the result of the spatial folding of the protein that actually configures a sequence similar to that from the former sequential epitope. They are also able to attach IgE, and are called ‘conformational epitopes’. This folding can be a consequence of different physic-chemical mechanisms such as hydrophobic interaction, electric bridges or di-Sulphur bridges. These conformational epitopes are quite common in proteins from whey milk, and they are very thermo-labile, which is why manufacturers of infant formulas use heat to induce thermal hydrolysis of proteins and avoid the attachment of specific IgE [45].

**Table 3:** The proteins of cow’s milk (original source: DRACMA guidelines [1])

Fraction	Protein	Allergen	g/L	% total protein	MW (kDa)	# AA	pI^a^
Caseins		*Bos d 8*	~30	80			
	Alpha_s1_-casein		12–15	29	23.6	199	4.9–5.0
	Alpha_s2_-casein		3–4	8	25.2	207	5.2–5.4
	Beta-casein		9–11	27	24.0	209	5.1–5.4
	Gamma_1_-casein		1–2	6	20.6	180	5.5
	Gamma_2_-casein				11.8	104	6.4
	Gamma_3_-casein				11.6	102	5.8
	Kappa-casein		3–4	10	19.0	169	5.4–5.6
Whey proteins			~5.0	20			
	Alpha-lactalbumin	*Bos d 4*	1–1.5	5	14.2	123	4.8
	Beta-lactoglobulin	*Bos d 5*	3–4	10	18.3	162	5.3
	Immunoglobulin	*Bos d 7*	0.6–1.0	3	160.0	-	-
	BSA^b^	*Bos d 6*	0.1–0.4	1	67.0	583	4.9–5.1
	Lactoferrin	-	0.09	traces	800.0	703	8.7

^a^Isoelectric point

^b^Bovine serum albumin

In the case of casein, containing mainly thermostable sequential epitopes, thermal hydrolysis is not sufficient. In this case, manufacturers use enzymatic hydrolysis, mainly with pepsin and trypsin, by which the protein is reduced to small fragments, with the aim of splitting the sequential epitopes. However, this splitting is blind and not epitope-selective so that it is possible that allergenic peptides persist.

The final process for the production of a hydrolysate is to ultra-filtrate the product in order to remove native whole proteins remaining, and fragments with high MW.

The results of these processes are the so-called eHFs. In these formulae, depending on the manufacturer, the size of the peptides varies. For example, in the case of whey or casein and whey formulas, Nestle declares that 0.3 % of the peptides contained in Alfaré® are between 2400 and 4000 Da, Nutricia that 4 % of peptides contained in Almiron® Pepti are greater than 3000 Da, Laboratorios Ordesa that 5 % of peptides of Blemil® Plus FH are between 1000 and 5000 Da, and so on. However, according to ESPHGAN, to meet the definition of eHF a product has not only to prove DBPCFC-negative in a proportion of CMA children greater than 90 % [46], but it is also required that:

- all Peptides have a MW lower than 5000 Da;
- a high proportion of them must have a MW even lower;
- the formula is uncontaminated with native whole proteins [47].

With such a definition, is complete avoidance (the must of CMA treatment) possible? Is a MW <5000 Da a sufficient warranty of non-exposure to cow’s milk proteins? Effector cells, as basophils and mast cells, express in their surface high-affinity receptors to which specific IgE attaches. When the density of IgE molecules attached to the cell membrane allows that a protein fragment containing two epitopes bypasses two of these IgE molecules, a release of the mediators contained in the cytoplasm occurs, and these are ultimately responsible for the allergic symptoms. On the other hand, it is well known that peptides formed by around 25 amino acids can contain two sequential epitopes, and peptides with only 10 AAs can include one sequential epitope [48]. A 1500 Da peptide may contain 11 amino acids, a 3000 Da peptide 22 and a 5000 Da peptide may contain 36 amino acids. Thus, peptides with a MW between 3000 and 5000 Da could possibly carry two sequential epitopes, whereas peptides with a MW of 1500 Da could contain one sequential epitope. A molecule carrying two epitopes could well determine an allergic reaction, while smaller fragments, containing only one sequential epitope, are not able to establish a bridge between two IgE molecules, and consequently the allergic reaction would not take place. However, are these fragments neutral, without any immunological effect? This is theoretically improbable, although there exists contradictory evidence in this sense. For example, an open non-randomized study suggests that peptides contained in eHF, even when tolerated by CMA children, can exert an immunomodulatory effect able to shorten the time for the acquisition of tolerance to CM [49], while a prospective randomized study reported the opposite. In this model, the median duration of CMA was 56 months when infants were fed an eHF, 28 months if they were fed a soy formula, and 20 months using a rice hydrolyzed formula [50]. Thus, *sensu stricto*, the use of eHFs does not completely avoid the causative protein. It can induce undesirable, sometimes severe reactions, and it can hypothetically contribute to the persistence of sensitization. There are no safe CM hydrolysates [51].

Other problems with eHFs may include palatability, cost, a higher solute renal load, and possible effects of a predigested formula in inducing delay in the intestinal enzymatic maturation. Similar problems may arise with elemental, amino-acid based formulae. Having said that, to avoid such problems, a completely different protein source (soy or rice) could be used.

## Soy formulae

Soy-based formulae are well tolerated in infants with IgE-mediated CMA but to a less extent in those with non-IgE mediated CMA. Many nutritional pitfalls with these formulae have been indicated in the past, the majority of which have been corrected by manufacturers.

Current soy formulas are supplemented with appropriate quantities of limiting amino acids such as Methionine, Taurine, and Carnitine [52]. They are not deficient in iron, zinc, calcium, phosphorus. The content in aluminum is more than 50 times greater in soy formulas than in breastmilk, but this is even truer for hydrolyzed formulas (80 times greater). However, 95 % of the ingested Aluminum is not absorbed in the gut and the kidney excretes the absorbed 5 %, so there are no differences in plasma aluminum levels in children fed with different formulas. Similar considerations are valid for Manganese. These two elements have been blamed for possible neurological damages, but no mental or developmental disorders have been detected among children fed with soy formulas as compared to cow’s milk formulas [53]. Soy formulae used to contain phytates which were blamed for their chelating capacity, preventing the appropriate absorption of several minerals and oligoelements. However, since the late eighties, phytates are almost totally removed from the soy formulae, resulting in substantially enhanced absorption of important micronutrients. Thus, a systematic review proved no significant differences in several biochemical parameters [54]. Raffinose and stachyose, responsible for bacterial fermentation and secondary flatulence, are nowadays removed from soybean products.

Two potential drawbacks remain for the use of soy formulas. One is the concern about possible hormonal effects on the reproductive system presumed due to phytoestrogens in the form of isoflavones (genistein, daidzen and their glycosides) present in soy protein. To date, the data do not support those concerns. The fertility and the general health of young adults has not been found to be affected by their exposure to soy formula as infants [55], and isoflavones have also been associated with a suppression of immune sensitization by suppressing Dendritic Cell (DC) maturation and its subsequent DC-mediated effector cell functions [56]. Masilamani et al. reported that dietary isoflavones significantly reduced the anaphylactic symptoms and mast cell degranulation in vivo after peanut challenge in a murine model of peanut allergy. Thus, they suggested dietary supplementation of soybean isoflavones as a possible novel strategy to prevent the development of allergic reactions to food [57]. The other problem to take into consideration is the use of transgenic soy in formulae. According to data from the US Department of Agriculture, up to 93 % of soybean crops are transgenic [58]. Although the available evidence suggests no deleterious effects on the human genome, reluctance to use transgenic food persists [59]. Due to these nutritional issues, ESPGHAN recommends not to use soy in infants with food allergy during the first 6 months of life [60].

## Rice hydrolised formulae

For all these reasons, a plausible alternative would be to use rice-based formulas. Rice is one of the less allergenic staple foods, reacting in <1 % of allergic children. It has no lactose and no phytoestrogens. For this reason, it has been developed as non-allergenic product in rice protein hydrolysates. These formulae are now in use for more than 10 years in Italy (Plasmon Risolac®, Heinz - Milan), in Spain (Blemil Plus Arroz Hidrolizado®, Laboratorios Ordesa - Barcelona), and in France (Modilac Expert Riz®, Sodilac – Paris; Novalac Riz®, Novalac - Paris). By enzymatic proteolysis, rice proteins have been hydrolyzed in the following ways:

- In Risolac®, 44 % of peptides have MW < 1000 Da, 43 % 1000–2000 Da, 13 % 2000–4000 Da;
- In Blemil Plus Arroz Hidrolizado® & Modilac Riz®, 96,6 % of peptides have MW < 5000 Da (26,8 % < 300 Da, 29,9 % with 300–1000 Da, 35,2 % 1000–5000 Da), and up to 10 % are free amino –acids [52]
- In Novalac Rice®, 95 % of peptides have MW < 1000 Da, and 99,4 % have MW ≤ 5000 Da [52].

The biological value of rice proteins is naturally different from bovine proteins: although they are rich in essential amino acids, three limiting essential amino-acids do not reach the respective value contained in breastmilk (Table 4). Based on partially hydrolyzed rice proteins supplemented with Lysine, Threonine, Tryptophan, Carnitine and Taurine, Iron and Zinc, RHFs are safe to children allergic to milk and soy [61] and to poliallergic infants [62]. A Spanish open, randomized clinical trial compared rice protein hydrolysate formula *(Blemil Plus Arroz Hidrolizado® 1 & 2)* versus casein hydrolysate *(Blemil Plus FH®)*. The 81 infants with CMA, median age 4.3 months, tolerated the formula in 100 % of cases [63]. To date, not a single case of reaction to an RHF is reported among children suffering from CMA.

**Table 4:** Essential or semi-essential amino-acids in rice vs. breastmilk (mg AA/g protein)

	Rice	Breastmilk
Arginine	83	38
Cysteine	18	13
Histidine	24	25
Isoleucine	43	40
Leucine	85	85
*Lysine*	36*	*67*
Methionine	37	16
Phenylalanine	55	34
*Threonine*	37*	*44*
*Tryptophan*	9*	*17*
Tyrosine	54	32
Valine	61	45

*Essential amino-acids not reaching the respective value contained in breastmilk

Their nutritional properties have been proved in several studies [54, 64] judged sufficient to warrant their safety by the metanalysis underlying the DRACMA guidelines [1]. Hydrolysate rice protein formulae supply 68–71 kcal/100 mL. Some alarming data on the nutritional effects of RHF were published 10 years ago. Eighty-eight infants with atopic dermatitis, 58 of whom with CMA confirmed at open challenge, were retrospectively evaluated. Fifteen were fed a rice-based hydrolysate formula (RHF), 17 a soy-based formula (SF), 26 an extensively hydrolysed casein formula (eHCF), and 30 infants with AD without cow’s milk allergy served as a control group (CG) [27]. No differences were recorded in weight for age during first 2 years of life between RHF, SF and eHCF group’s z-score. The group fed RHF showed a reduction in weight vs control group in the age intervals 9–12 months (*p* = 0.025) and 12–18 months (*p* = 0.020). By contrast, SF and eHCF groups were comparable to the control group, except for the 1st trimester of life (eHCF group significantly lower). The authors concluded that these retrospective data pose some questions on the nutritional adequacy of rice-hydrolysate formulas.

More reassuring information came from later prospective studies. A prospective clinical assessment of tolerance to a rice-based hydrolysed formula was carried out in 100 children allergic to cow’s milk [54]. All patients were sensitized to cow’s milk and/or at least one cow’s milk protein fraction and CMA was confirmed at double blind, placebo-controlled food challenge (DBPCFC) when not contraindicated. DBPCFC was carried out with increasing doses of a rice-based hydrolysed formula and all children tolerated it. Another prospective study assessed growth adequacy between 6 and 12 months in 93 children with IgE-mediated CMA, breastfed at least 4 months and weaned at 5–6 months. Infants were randomized to three types of feeding formula:

- Soy (32 infants; Isomil 2®)
- Casein hydrolysate (31; Nutramigen®)
- Rice protein hydrolysate enriched in lysine and threonine (30; Risolac 2®).

A reference group, non-randomized, was made of infants with CMA breastfed until 12 months (*n* = 32). All groups displayed low weight/age z-score at enrolment (6 month); this was interpreted as an effect of CMA. Infants fed casein or rice protein hydrolysates had higher z-score weight/age than infants fed soy at 9 and 12 months. A quicker weight catch-up was found with RHF than with eHF at 9 and 12 months. Infants fed rice hydrolysate showed height/age z-score identical to those fed soy and those breastfed at 9 and 12 months. Infants fed casein hydrolysate had a higher height/age z-score at 9 and 12 months. These data showed that rice hydrolysate ensured an appropriate growth [28].

More data on this aspect came from the Spanish study mentioned above [55]. At enrolment, all infants had a weight below the mean to Spanish reference, probably due to CMA (as already seen in [28]). Weight for age, height for age, and weight for height did not differ among RHF- and eHF-fed children.

In a French study, 78 healthy term infants, <1 month old, were exclusively fed hydrolyzed rice protein formula (Modilac Riz®) until the introduction of solid foods in an open-label, multicenter study. The change in the formula was done because of digestive troubles (colics, gas, and regurgitation) or risk of allergy. Infants presenting a cow’s milk protein allergy were excluded. Their daily weight gain over 5 months was 23.2 ± 4.3 g/day, identical to WHO standards (22.2 ± 1.8 g/day, *P* = 0.09). The Z-scores for weight, height, and BMI varied between +1.1 and −0.5 SD according to the WHO standards. Formula acceptance and tolerance were both good [65]. Last, a prospective trial assessed clinical tolerance of eRHF [30]. Forty infants (mean age, 3.4 months; range, 1–6 months) with CMA confirmed by a food challenge tolerated the eRHF with a symptom score significantly decreased from the first month of intervention on. Those fed eRHF showed a catch-up to normal weight gain from the first month, and a normalization of the weight-for-age, weight-for length, and BMI z-scores within the 6-month study period. This study concluded that eRHF was tolerated by more than 90 % of children with proven CMA with a 95 % confidence interval, indicating it as an adequate and safe alternative to cow milk-based eHF.

From these studies, the allergologic and nutritional safety of the RHFs is clear. Where available, these products could become the first line treatment of CMA even in severe forms.

## Conclusions

Six years after the publication of DRACMA guidelines, new science in breastfeeding makes even clearer that its benefits are inimitable. In the case of CMA, every effort should be done to preserve the breastfeeding. When this is not possible, many options are available. Although we provided a review of some points of interest, our indications will not necessarily translate in to different recommendations after a review of the DRACMA metanalyses. To our understanding, however, some points have to be taken into account:

- non IgE-mediated CMA should be better defined;
- First diagnosis and management should fall under the DRACMA recommendations;
- The dietetic management of CMA depends on the type of allergic reaction;
- CMP-based eHF may not guarantee a complete avoidance;
- The possible tolerogenic role of exposure to milk protein residues in eHF must be taken in account;
- Nutritional, economic, and allergologic aspects should be appropriately balanced.

The accruing evidences on diagnosis and treatment now make an update of the guidelines advisable. Between 2010 and 2016, DRACMA modified the diagnostic and prescriptive attitudes of pediatricians [66], and the guidelines may be modified by the new evidences. Following the strictest criteria for EBM, and using the Grading of Recommendations Assessment, Development and Evaluation (GRADE) approach, the updated DRACMA aims to answer more clinical questions, adhering to the needs of pediatricians worldwide.

## Abbreviations

AA: Arachidonic acid
AAF: Amino acid formulae
CM: Cow’s milk
CMA: Cow’s milk allergy
CNS: Central nervous system
DC: Dendritic cell
DHA: Docosahexaenoic acid
DRACMA: Diagnosis and Rationale for Action against Cow’s Milk Allergy guidelines
eHF: extensively hydrolyzed formulae
EPA: Eicosapentaenoic acid
ESPHGAN: European Society for Pediatric Gastroenterology and Nutrition
GRADE: Grading of Recommendations Assessment, Development and Evaluation
MW: Molecular weight
PUFA: Poly-unsatured fatty acids
rHF: rice hydrolyzed formulae
SF: Soy formulae
